# Efficacy of *Juglandis semen* complex extract for knee osteoarthritis: A pilot study protocol for a 12-week, single-center, randomized, controlled, double-blind clinical trial

**DOI:** 10.1097/MD.0000000000016956

**Published:** 2019-08-23

**Authors:** Hae Jin Kong, Jae Hui Kang

**Affiliations:** a Department of Acupuncture & Moxibustion Medicine, College of Korean Medicine, Daejeon University, Daejeon, South Korea.

**Keywords:** *Juglandis semen*, knee osteoarthritis, protocol, randomized controlled trial

## Abstract

**Background:**

Knee osteoarthritis (KOA) is a common disease in elderly individuals. Many medications for KOA have the potential to cause side effects. We used *Juglandis semen* complex extract (JCE) consisting of 4 herbs derived from Cheong-A-Won, which has been commonly used for KOA treatment. In this study, we will evaluate whether JCE improves symptoms in patients with KOA and will identify the changes in the inflammation factor.

**Methods:**

This study will be a single-center, randomized, double-blind, and placebo-controlled trial. Three groups, JCE 1000 mg, 2000 mg, and placebo, will be randomly allocated. Total duration of the clinical trial will be 12 to 14 weeks. Study participants will be followed up every 6 weeks and the effect and safety will be assessed at the 2^nd^, 3^rd^, and 4^th^ visit. All participants were asked to maintain a dosage schedule for this protocol. The primary outcomes will be measured using Korean Western Ontario and McMaster Universities Questionnaire and the secondary outcomes will include pain Visual analog scale score, EuroQol Five Dimensions questionnaire, Patient Global Impression of Change, and the changes in the laboratory test parameters of inflammation. Repeated-measure analysis will be used to measure primary efficacy based on full analysis set.

**Discussion:**

This study has limited inclusion and exclusion criteria and a well-controlled intervention, and it will be the first randomized controlled trial to assess the efficacy and safety of JCE in patients with KOA. This study provides insights into the mechanisms that explain the therapeutic effects of JCE in KOA and will lay the groundwork for further studies.

## 1 Introduction

Knee osteoarthritis (KOA) is a major cause of knee joint pain and is very common in elderly individuals. The symptoms of KOA are characterized by pain, inflammation, and stiffness in the musculoskeletal system, which ranges from localized self-limiting conditions to systemic autoimmune processes.^[1]^

The treatment of KOA is divided into conservative and surgical treatment. Basically, the symptom of pain is managed by conservative treatments such as hyperthermia, exercise therapy, and medication. However, if there is severe pain without improvement, joint deformity, instability, and limitation of movement, then surgical treatment such as osteotomy, arthroscopic surgery, and artificial joint replacement should be considered.^[2]^

In this study, we will try to prepare *Juglandis semen* complex extract (JCE) from *Juglandis semen*, *Eucommia ulmoides*, *Acanthopanax sessiliflorum*, and *Zingiber officinale*. This extract is referred to as “Cheong-A-won,” which was first recorded in “Tae-pyung-hye-min-hwa-je-kook-bang.” “Cheong-A-Won” is composed of *Juglandis semen*, *Eucommia ulmoides*, *Psoralea corylifolia*, and *Zingiber officinale*. Among these, *Psoralea corylifolia* is effective in promoting osteoblast proliferation and has been widely used in bone diseases.^[3]^ However, in this study, *Acnthopanax sessiliflorum*, safe for use in humans and widely used in conditions of the musculoskeletal system, was used instead of *Psoralea corylifolia* because there is a controversy about liver damage due to a toxic component of it.^[4]^ In the present study, the protocol was designed to verify the efficacy of JCE for symptoms of patients with KOA using a single-center, randomized, controlled, and double-blind clinical trial.

## 2 Methods

### 2.1 Study design

**Figure 1:**
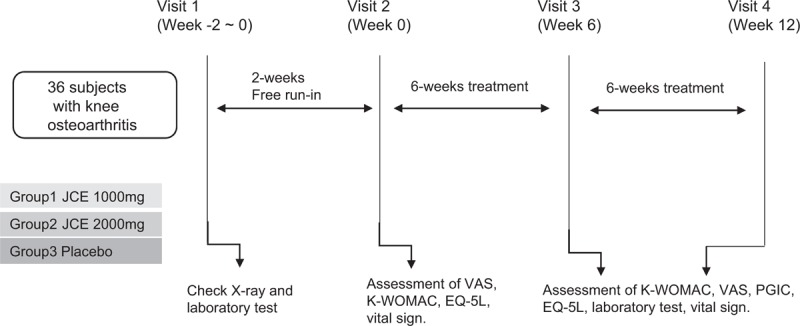
Study design for JCE clinical trial. The treatment or control groups will be administered JCE 1000 mg or JCE 2000 mg or placebo. The subjects will receive JCE 1000 mg or JCE 2000 mg or placebo for 12 weeks and will visit every 6 weeks. JCE = *Juglandis semen*.

This study has been designed as a double-blind, single-center, randomized clinical trial for investigating the efficacy of JCE in KOA. A total of 36 subjects with KOA will be recruited from outpatients at the Daejeon University Cheonan Korean Medicine Hospital through advertisements posted on bulletin boards at hospitals, apartments, and so on. Recruitment commenced in April 2019, and the trial is expected to end in October 2019. All participants will receive a full written explanation of the study's protocol and an informed consent form. x-ray imaging of both knees will be performed for those who are suspected to have KOA based on history or physical examination. Additionally, blood tests for blood clotting factors, liver function, inflammation-related enzymes, and complete blood count will be performed. A total of 36 patients with knee pain who meet the eligibility criteria for this study will be randomly assigned in a 1:1:1 ratio to the treatment group 1 (JCE 1000 mg), treatment group 2 (JCE 2000 mg), or control group (placebo). The intervention will begin within 2 weeks of the screening visit. Based on the study group, the subjects will be administered JCE 1000 mg, JCE 2000 mg, or placebo for 12 weeks. The total duration of the clinical trial will be 12 to 14 weeks. Subjects will be followed up every 6 weeks, and the efficacy and the safety will be assessed at the 3^rd^ and 4^th^ visits. The study flow chart is presented in Figure 1.

### 2.2 Inclusion and exclusion criteria

The inclusion criteria are as follows: age, 40 to 75 years; knee pain for ≥6 months; grade 1 or 2 on the Kellgren-Lawrence grading scale on both knee x-ray examinations; whether the subject is taking arthritis medication such as non-steroidal anti-inflammatory drugs (NSAIDs), those who have not changed their medication within the last a month.

The exclusion criteria are as follows: KOA owing to factors like gout and rheumatism other than degenerative; history of fractures of the lower limb in the last 3 months; intra-articular injection of hyaluronic acid or steroids within 3 months of the study; continuous intake, 1 month before the study, of steroids or NSAIDs that may affect KOA; severe gastrointestinal symptoms such as heartburn and indigestion; alcohol abuse; clinically significant cardiovascular, immune, infectious, and/or neoplastic diseases other than arthralgia; suspected liver, kidney, or blood disease; habitual administration of psychotropic drugs or narcotic analgesics that may interfere with pain sensation; pregnancy or breastfeeding; food allergies to nuts, and so on; and participation in the other clinical trials within 30 days.

### 2.3 Sample size calculation

We have demonstrated both in vitro and in vivo experiments that JCE has improved KOA and has antioxidant effects. To determine the minimum effective concentration, JCE was administered at various concentrations, and 100 mg/kg and 200 mg/kg of JCE was administered to the mice for 14 days, based on which it was confirmed that JCE had an excellent effect on improving the joint symptoms. Based on the US FDA guidelines for calculating the effective dose according to the body surface area in humans, the effective dose in the animal tests was calculated as the effective dose in terms of human body. Based on these calculations, the dose in the human body was set at 1000 mg and 2000 mg. Julius suggests that for the preliminary study and parallel design, the empirically optimal sample size is 12 persons per group, considering feasibility, estimated average, and dispersion accuracy.^[5]^ Therefore, we designed a pilot study with 12 subjects for each group.

### 2.4 Randomization and blinding procedures

Randomization will be performed by stratified block randomization method in experimental groups 1 and 2 and control group at a ratio of 1:1:1 to ensure a balanced distribution of the 3 groups. Subjects who meet all registration criteria will be randomly assigned to randomization identification code (ex. KOA-R-001, KOA-R-002, …, KOA-R-036) in the order generated by a computer randomized program. The randomization identification code will indicate whether JCE 1000 mg, 2000 mg, or placebo is to be given. As this pilot study is designed to be double-blinded, the participants, researchers, and assessors collecting the data will be blinded to the group allocation.

The information of intervention assignment will be stored in the third statistical department. The randomization code will be placed in an opaque envelope and stored at the hospital. With the exception of disclosure to individual patients in emergency situations, randomization and blinding were not disclosed to researchers until the end of the trial.

### 2.5 Intervention

All eligible participants will receive a treatment according to the allocated group, JCE 1000 mg, 2000 mg, or placebo, in the following 12-week treatment period. To maintain the double-blinded nature of the study, JCE 1000 mg, 2000 mg, and placebo will be manufactured to be similar in appearance, smell, and taste, and will be indistinguishable to which group the clinical trial subjects assigned. The subjects will be evaluated twice in 12 weeks (at 6 and 12 weeks). JCE 1000 mg, JCE 2000 mg, and placebo will be manufactured by Hankookshinyak Pharmaceutical Co. (Nonsan, Republic of Korea) and 2 sticks will be taken orally twice a day.

### 2.6 Outcome measures

The primary outcome is Korean Western Ontario and McMaster Universities Questionnaire (K-WOMAC) score, which will be used to assess items related to knee pain, joint stiffness, and physical functioning in the 6^th^ and 12^th^ week of treatment. The secondary outcomes are pain visual analog scale score after 6 and 12 weeks of treatment, EuroQol Five Dimensions questionnaire survey score for rating quality of life, Patient's Global Impression of Change on treatment and the change of erythrocyte sedimentation rate, and C-reactive protein level, which indicated inflammation in the body. For safety of the subjects, we will conduct laboratory tests such as blood clotting factors, liver function, inflammation-related enzymes, and complete blood count, and evaluate vital signs. Safety assessments will be conducted at every visit. Participants’ data will be anonymized and coded by a specific program.

### 2.7 Data collection and monitoring

During the screening period, the subjects will complete a questionnaire regarding their sociodemographic characteristics, provide their medical history, take a x-ray of both knee, and perform the laboratory test. Personal information and data collected during the screening period will be shared and managed by the hospital. The monitoring of data and research performances will be conducted regularly by researchers from the Jeneolurl Bio Taek Co., Ltd. (Pusan, Republic of Korea). The final trial dataset is accessible to statisticians and principal investigator. The results of this study will be published in a peer-reviewed article.

### 2.8 Statistical analysis

Statistical analysis will be primarily based on the principle of full analysis set. The missing values will be analyzed by the last observation carried forward method. For each of the 2 experimental groups and the control group, the baseline values before treatment and the changes in K-WOMAC at 6 and 12 weeks after treatment will be measured, and the descriptive statistics will include measurement of mean, standard deviation, median, minimum, and maximum. The analysis method for evaluation of the variables of efficacy involves confirmation of the normality of the values and then comparison using analysis of variance or Kruskal–Wallis test and post-test using Bonferroni method. The significance level will be set at *P* < .05 and the confidence interval at 95%. All statistical analyses will be performed using SPSS Statistics for Windows Version 20.0 (IBM Corp., Armonk, NY).

If necessary, subgroup analyses of outcome variables such as demographic variables measured at baseline (eg, sex, age, duration of illness, treatment expectation, and so on) can be performed. Safety assessments will be performed primarily by analyzing the frequency of adverse events and severe adverse events (SAEs) that researchers suspect to be related to treatment. The collected safety data will be summarized appropriately. All adverse events that occur will be listed with a detailed description. All SAEs will be recorded descriptively. The adverse events will be collected through patient symptom reporting and researcher observation. The frequency of each adverse event associated with or unrelated to the intervention will be recorded and analyzed using descriptive statistics. There is no intermediate analysis of this study, and the final decision regarding the completion of the clinical trial will be with the principal investigator.

### 2.9 Withdrawal and dropout

If the subject does not meet the inclusion or exclusion criteria or withdraws his/her consent, or if the subject's continued participation is judged as inappropriate, the subject will be excluded from the study. The researchers will record the reason for any interruption in the intervention and whether each participant completed the study.

### 2.10 Safety

The occurrence of all adverse events will be assessed at each visit. Subjects will be monitored for undesirable, unintended symptoms, signs, and illnesses. The number and percentage of subjects who experience at least 1 adverse event will be calculated.

### 2.11 Ethics

This study is designed based on the Helsinki Declaration and the Korean Clinical Practice Guidelines and has been approved by the Korean Institutional Review Board of DUCKMH (number DJUMC-2018-BM-09-3). This study protocol is registered with the Korean National Clinical Research Information Service (CRIS) (CRIS-KCT0003552). Subjects may be required to quit the study in case of serious adverse events or adverse drug reaction, and then they will be reported to institutional review board in a hospital. All participants of this study will be allowed to withdraw their consent at any time for any reason or discontinue their participation on a voluntary basis.

## 3 Discussion

KOA is a chronic degenerative disease in which the articular cartilage is worn or damaged and clinically manifests as joint dysfunction owing to pain, stiffness, deformation, and edema of the joint tissue.^[6]^ Although the exact cause has not been elucidated yet, it is considered to be a major risk factor in wear of the joint because of aging and degeneration of the cartilage.^[7]^

Recently, KOA treatment has been aimed at relieving symptoms by reducing pain and edema. NSAIDs are frequently used as medications to reduce the inflammatory response.^[8]^ Treatments include drug therapy, physical therapy, surgery, and injection therapy.^[9]^ As KOA is a chronic disease, most people take NSAIDs for a long time, which often causes gastrointestinal side effects and nephrotoxicity.^[10]^ For these reasons, there is a growing interest in herbal medicines that are effective and safe in KOA.

*Juglandis semen* has been reported in various studies for its antioxidant and analgesic effects.^[11]^ Additionally, *Eucommia ulmoides* shows antioxidant activity and has anti-inflammatory effect.^[12,13]^*Acanthopanax sessiliflorum* is known to be effective for neuralgia, joint pain, and bruises.^[14]^ Further, *Zingiber officinale* is known to have anti-inflammatory and antioxidant effects.^[15]^ The purpose of this study is to investigate the efficacy of the combination of 4 herb medicines in KOA.

This is the first study at this stage to be conducted to determine the appropriate dosage and duration of JCE administration. However, the sample size may be small, and thus, a bias may occur. However, this study may provide insights into the mechanisms underlying the therapeutic effects of JCE in KOA and can lay the foundation for further studies on the effect of JCE on symptoms in patients with KOA.

## Author contributions

**Conceptualization:** Jae Hui Kang.

**Data curation:** Haejin Kong.

**Formal analysis:** Haejin Kong.

**Funding acquisition:** Jae Hui Kang.

**Investigation:** Haejin Kong, Jae Hui Kang.

**Methodology:** Haejin Kong, Jae Hui Kang.

**Project administration:** Jae Hui Kang.

**Resources:** Jae Hui Kang.

**Software:** Haejin Kong.

**Supervision:** Jae Hui Kang.

**Validation:** Haejin Kong.

**Visualization:** Haejin Kong.

**Writing – original draft:** Haejin Kong.

**Writing – review & editing:** Haejin Kong, Jae Hui Kang.

HAEJIN KONG orcid: 0000-0002-6737-8564.
